# Chondroitin Sulfate Induces Depression of Synaptic Transmission and Modulation of Neuronal Plasticity in Rat Hippocampal Slices

**DOI:** 10.1155/2015/463854

**Published:** 2015-05-13

**Authors:** Elisa Albiñana, Javier Gutierrez-Luengo, Natalia Hernández-Juarez, Andrés M. Baraibar, Eulalia Montell, Josep Vergés, Antonio G. García, Jesus M. Hernández-Guijo

**Affiliations:** ^1^Teófilo Hernando Institute for Drug Discovery, Facultad de Medicina, Universidad Autónoma de Madrid, 28029 Madrid, Spain; ^2^Department of Pharmacology and Therapeutics, Facultad de Medicina, Universidad Autónoma de Madrid, 28029 Madrid, Spain; ^3^Department of Clinical Pharmacology, Instituto de Investigación Sanitaria Hospital de la Princesa, Universidad Autónoma de Madrid, 28029 Madrid, Spain; ^4^Departamento de Investigación, Laboratorios Bioibérica, 08029 Barcelona, Spain

## Abstract

It is currently known that in CNS the extracellular matrix is involved in synaptic stabilization and limitation of synaptic plasticity. However, it has been reported that the treatment with chondroitinase following injury allows the formation of new synapses and increased plasticity and functional recovery. So, we hypothesize that some components of extracellular matrix may modulate synaptic transmission. To test this hypothesis we evaluated the effects of chondroitin sulphate (CS) on excitatory synaptic transmission, cellular excitability, and neuronal plasticity using extracellular recordings in the CA1 area of rat hippocampal slices. CS caused a reversible depression of evoked field excitatory postsynaptic potentials in a concentration-dependent manner. CS also reduced the population spike amplitude evoked after orthodromic stimulation but not when the population spikes were antidromically evoked; in this last case a potentiation was observed. CS also enhanced paired-pulse facilitation and long-term potentiation. Our study provides evidence that CS, a major component of the brain perineuronal net and extracellular matrix, has a function beyond the structural one, namely, the modulation of synaptic transmission and neuronal plasticity in the hippocampus.

## 1. Introduction

In the central nervous system the extracellular matrix forms a compact and organized matrix called perineuronal nets (PNNs) [1, 2]. The composition of this net is unique and different from of other tissues. CS proteoglycans (CSPGs) are the main component of perineuronal nets [3–5] and consist of a large variety of core proteins covalently linked to chondroitin sulfate glycosaminoglycans (CS-GAGs) [6]. Astrocytes, neurons, oligodendrocytes, and microglia seem to synthesize the CSPGs participating in the formation of perineuronal nets [7]. The role of PNNs is not clear; it is currently known that they are involved in synaptic stabilization and limitation of synaptic plasticity [8, 9]. Due to the negative charge of CS-GAGs, they can bind to various cations, as sodium, potassium, and calcium, acting as a kind of buffering system [10–12]. In Alzheimer's disease, it has been reported that cortical areas highly rich in perineuronal nets are less affected by degeneration [13].

After CNS injury, a glial scar is formed [14–16] to reestablish the integrity of the CNS [17–19]. This enhances the CS-PGs within the glial scar which remain in the injured sites for a long period of time [20, 21] whereby they are considered to inhibit neural regeneration [22, 23]. The inhibitory activity of CS-PGs is mediated mainly by the CS-GAGs [24]. The molecular mechanisms involved in the inhibitory effects of CS-GAGs are still not clear. They could be related to a direct interaction with a CS-GAG receptor [25, 26] or through binding to specific growth inhibiting elements [27]. Alternatively, their effects can be mediated through the control of CS-GAGs and integrins activation, although a direct interaction between CS-GAGs and integrins has not been demonstrated [28]. Other studies have shown that the function of CSPGs may be related to the specific sulfation sequence of CS chains [29–32] that could act as both inhibitory molecules for axonal growth and neuritogenic molecules [18, 32]. In line with this is the observation that the treatment with chondroitinase following injury allows the formation of new synapses, increases synaptic plasticity, and leads to functional recovery [33–36].

On the other hand, some postsynaptic neurotransmitter receptors are also directly influenced by components of the extracellular matrix [37]. It has been reported that the removal of hyaluronic acid from hippocampal slices suppresses L-type Ca^2+^ currents and reduces Ca^2+^ transients [38]. One of the primary events for the induction of long-term potentiation (LTP) at this synapse is the elevation of postsynaptic Ca^2+^ concentrations [39]; so, these molecules may modulate neuronal plasticity [38]. Thus, the study of the role of perineuronal net components on synaptic transmission is important to understand both neuronal excitability and plasticity [40]. Other studies have also reported that removal of hyaluronic acid facilitates lateral diffusion of membrane molecules, including AMPA receptors, and reduces the level of paired-pulse depression [38, 41]. It is also known that digestion of CS-PGs with chondroitinase impairs early-LTP (e-LTP) [42] and mice deficient in the CS-PGs brevican or neurocan show impaired e-LTP and late-LTP (l-LTP), respectively [43]. We have recently reported that CS induces Na^+^ inward current that causes cell depolarisation and a transient elevation of the cytosolic Ca^2+^ concentration ([Ca^2+^]_c_) in cultured rat hippocampal neurons; these effects were selectively mediated by AMPA/kainate receptors [44].

Our present study provides evidence that, in addition to playing an important role in the structural integrity of mammalian extracellular matrix, CS can also modulate cellular excitability, synaptic transmission, and neuronal plasticity in rat hippocampal slices. Thus, CS caused a reversible depression of synaptic transmission that was not prevented by an antagonist of kainate receptors. On the other hand, CS decreased the population spike mediated by synaptic transmission but enhanced the population spike evoked antidromically. Additionally, CS exhibited a modulator role in neuronal plasticity by enhancing both paired-pulse facilitation and e-LTP. Our data strongly suggest that not only CS has a structural function in the CNS but also this GAG has functional effects, for instance, the modulation of synaptic transmission.

## 2. Materials and Methods

### 2.1. Preparation of Hippocampal Slices

All experiments were performed on 400 *μ*m thick transverse hippocampal slices obtained with standard methods from male Sprague-Dawley rats (200–250 g). The care and use of animals were carried out in accordance with the National Council on Animal Care and the European Communities Council Directive and were approved by the local Animal Care Committee of Universidad Autónoma de Madrid. Animals were decapitated after anaesthesia with isoflurane, and the brain was quickly removed and dropped into ice-cold Krebs-Ringer bicarbonate (KRB) solution containing (in mM) the following: 119 NaCl, 26.2 NaHCO_3_, 2.5 KCl, 1 KH_2_PO_4_, 1.3 MgSO_4_, 2.5 CaCl_2_, and 11 glucose. This solution was gassed with 95% O_2_ and 5% CO_2_. The hippocampi were sliced with a manual tissue chopper and placed in an interface holding chamber for at least 2 h at room temperature (20–25°C). A single slice was transferred to a submersion-type recording chamber where it was continuously superfused (2 mL/min) with standard KRB solution. Bath temperature in the recording chamber was maintained at 31-32°C.

### 2.2. Extracellular Recordings of Evoked Synaptic Potentials

Synaptic responses were evoked by stimulating the collateral-commissural fibbers of Schaffer with electrical pulses (20–50 *μ*A, 100 *μ*s, 0.033–0.066 Hz) applied through bipolar tungsten insulated microelectrodes placed on CA1* stratum radiatum*. This stimulating pulse evoked a fEPSP of about 1 mV amplitude that represents the 40–50% of maximal response. Electrical pulses were supplied by a stimulus isolation unit (Cibertec, Model ISU200BIP, Madrid, Spain). The field EPSP and the presynaptic fibber volley (FV) from the* stratum radiatum* of the CA1 region were recorded with tungsten microelectrodes (1 MΩ) connected through a home-made preamplifier to a Grass amplifier (Model 7P511H).

### 2.3. Electrophysiological Data Analysis

Evoked responses were low-pass-filtered at 3 kHz and digitized at 25 kHz using a Digidata 1440A board (Axon Instruments) and stored on a computer using pCLAMP-10 software (Axon Instruments). The amplitude of the presynaptic FV was measured from the baseline to the negative peak of the FV. The synaptic strength was calculated using the initial slope phase (1 ms window) of the fEPSP to avoid the possible contamination of the response by propagated population spikes. Field EPSP slope is considered a fine indicator of glutamatergic synaptic transmission. As baseline of field potentials we took the mean value of the signal (2–5 ms) preceding the stimulus artefact. We used pCLAMP-10 software for these calculations. Traces shown are averages of eight consecutive responses. Data were normalized with respect to the mean values of the responses at baseline period in standard medium.

### 2.4. Chemicals

Chondroitin sulfate used in the present study was provided by Bioibérica (Barcelona, Spain). CS is highly purified chondroitin sulfate of bovine origin in a concentration above 98% (measured by CPC titration assay, the official assay method of the USP CS monograph and European Pharmacopeia to ensure a correct measure of CS purity and potency); however, an effect due to unknown components that constitute the remaining 2% cannot be ruled out. This product from Bioibérica consists of a mixture of CS sulfated in positions 4 (62%), 6 (32%) or unsulfated (6%) on the N-acetyl-D galactosamine group. The full range of its molecular weight is ~13–16 kDa with an intrinsic viscosity of ~0.02–0.06 m^3^/Kg. (RS)-1-(2-Amino-2-carboxyethyl)-3-(2-carboxybenzyl)pyrimidine-2,4-dione (UBP-296) was supplied by Tocris Bioscience (Bristol, United Kingdom). CS-4S (cat. number 27042), heparin (cat. number H3393), and other chemical components for solutions were obtained from Sigma-Aldrich (Madrid, Spain).

Chondroitinase ABC from* Proteus vulgaris* (cat. number C3667, Sigma-Aldrich, Madrid, Spain) was prepared (50 mU/mL) in a modified KRB (50 mM sodium acetate as reaction activator was added).

The drugs were prepared as stock solutions stored frozen in the dark and diluted to the final concentration in the superfusion solution immediately before use. The osmolarity of the superfusion solutions was tested with a microosmometer (Advanced Instruments, Model 3MO, Norwood, MA, USA).

### 2.5. Statistical Analysis

Data were expressed as means ± SEM of the number of slices (*n*) studied, from at least 3 different animals. Student's *t*-test was used to determine statistical differences between means. The level of statistical significance between two groups of data was established at *p* < 0.05.

## 3. Results

### 3.1. Chondroitin Sulfate Reversibly Reduces Synaptic Efficacy in Rat Hippocampal Slices

**Figure 1:**
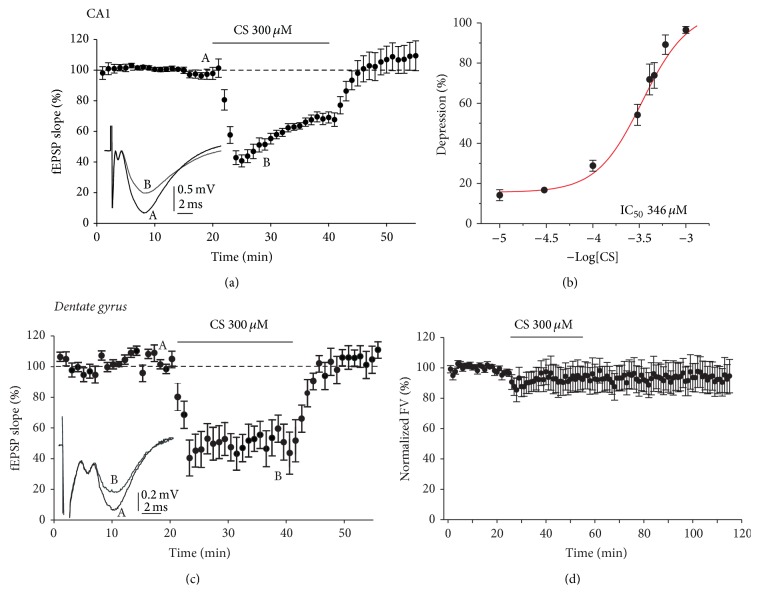
Chondroitin sulfate (CS) reversibly depresses synaptic transmission in the hippocampal slice. (a) Time-course of fEPSP slope depression exerted by CS (300 *μ*M) applied during the time indicated by the top horizontal bar. Every dot represents the minute-averaged fEPSP slope. Note the fast turn-on and turn-off of effects exerted by CS. Insets A and B represent the original traces of fEPSPs in the points A and B of the time-course curve. (b) Degree of depression caused by each concentration of CS at the end of 20 min of perfusion. A single concentration of CS per slice was tested. Data were fitted to the concentration-response curve. Data represent the mean ± SEM (*n* = 4 to 8 slices). (c) Time-course of fEPSP slope in the* dentate gyrus* measured before, during, and after CS applied during 60 min (top horizontal bar). Inset, original traces taken from points A and B of the time-course curve. (d) Time-course of changes on fibber volley (FV) amplitude induced by bath application of 300 *μ*M CS for the time indicated by the top horizontal bars. Data were normalized to the mean value of the control period and expressed as mean ± SEM/min of 8–13 experiments.

Initially, we determined the effect of different CS concentrations (from 10^−5^ to 10^−3^ M) on glutamatergic synaptic transmission in the hippocampal CA1 area. Only a single concentration of CS was applied to each individual slice. Once a stable fEPSP baseline was established for 20 min, CS was applied in the perfusion liquid causing a fast dose-dependent reduction of fEPSP (Figure 1). When CS was washed out, synaptic responses returned to baseline values in about 5 min (Figure 1(a)). The magnitude of depression observed during the last 2 min of CS application was fitted to a sigmoid function which gave an IC_50_ of 346 *μ*M (Figure 1(b)). At the highest concentration of CS (1 mM), the fEPSP showed a maximal depression of 88.7 ± 3.5%. We selected the concentration of 300 *μ*M CS applied for 20 min to perform all subsequent experiments.  Figure 1(c) shows the time-course of CS effect exerted on synaptic responses evoked in* dentate gyrus*. The effects mediated by CS were similar in both hippocampal regions, that is, CA1 and* dentate gyrus*.

Changes in the number of axons recruited by electrical stimulation will elicit changes in the same direction in the number of synaptic terminals releasing glutamate which, consequently, will modify synaptic potential size. Hence, we wondered whether the reduction in fEPSP caused by CS was due to the decrement of fibres activated by the stimulus in the presence of this drug. To answer this question, we recorded the fibber volley (FV) that corresponds to the number of axons that were firing action potentials synchronously [45]. The application of CS reduced the fEPSP slope (Figures 1(a) and 1(c)) but did not affect significantly FV amplitude (Figure 1(d)). So, these results revealed that CS-induced fEPSP decrease was not due to reduction in the number of recruited axons, representing therefore a reduction in synaptic efficacy. We have used a commercial form of CS-4S that did not mimic the effect obtained by CS from Bioibérica.

### 3.2. Chondroitin Sulfate Application Modifies Synaptic Facilitation Induced by Paired Pulses

We next attempted to clarify whether CS-induced fEPSP reduction involves a presynaptic alteration of release mechanisms that could be detected with the stimulation paradigm of paired-pulse facilitation (PPF) [46].

**Figure 2:**
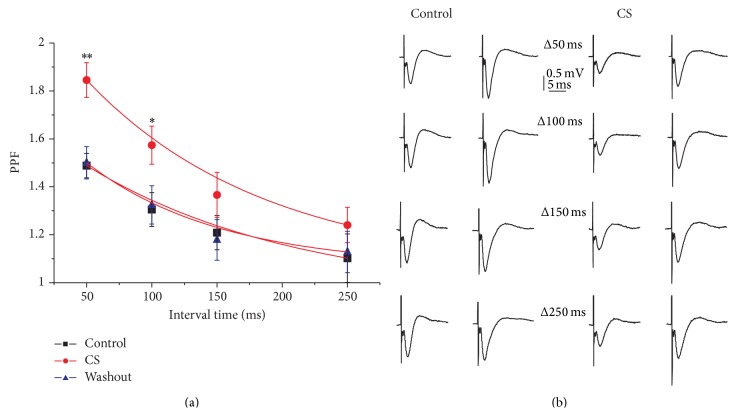
Effect of CS on paired-pulse facilitation (PPF). To examine if CS might affect presynaptic glutamate release, PPF was measured by calculating the percent facilitation of the second pulse fEPSP over the first pulse (fEPSP_2_ slope/fEPSP_1_ slope). (a) The mean PPF was plotted as a function of the interpulse interval and fitted by an exponential function. (b) fEPSPs recorded in response to paired stimuli separated by the indicated interpulse intervals (Δ*t*) in control (left) and after 20 min of CS application (300 *μ*M) (right). The fEPSP amplitude evoked corresponds to the 40–50% of maximal response. Data are mean ± SEM of 10 slices. ^∗^ *p* < 0.05, ^∗∗^ *p* < 0.01.

**Figure 3:**
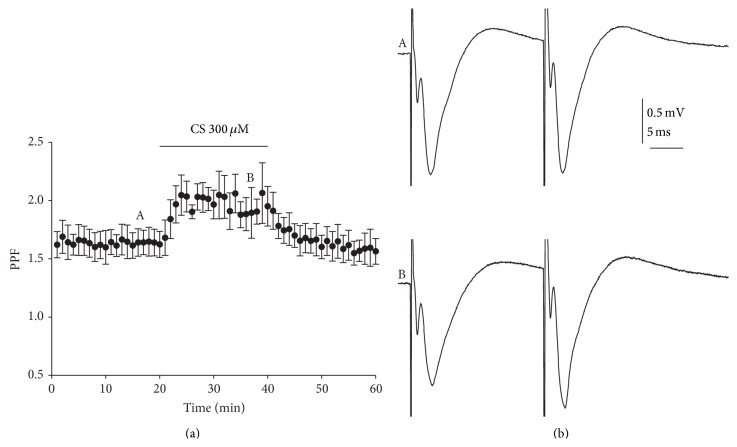
Presynaptic effect of chondroitin sulfate. (a) Time-course of averaged facilitation of fEPSP evoked by paired pulses at 50 ms intervals and applied every 15 s. Note the facilitation exerted by CS (300 *μ*M) applied during the time indicated by the top horizontal bar. (b) Representative fEPSPs recorded in response to paired stimuli separated by 50 interpulse intervals in control A and after 20 min of CS (300 *μ*M) application B. The amplitudes evoked correspond to the 40–50% of maximal response. Data are mean ± SEM of 14 slices evaluated.

Pairs of synaptic responses were evoked by two stimuli with an interstimulus interval of 50–250 ms applied every 15 s throughout the experiment. This stimulation pattern induces, for a short time, an increase in transmitter release resulting from the residual presynaptic free Ca^2+^ levels that is more evident at the shorter stimulus intervals. As shown in Figure 2 CS mainly decreased the first pulse but did not affect the second fEPSP; therefore, the normalized facilitation ratio (second pulse/first pulse) changed during CS perfusion at all intervals evaluated (1.85 ± 0.07, 1.57 ± 0.08, 1.36 ± 0.09, and 1.24 ± 0.07) when compared with control conditions (1.48 ± 0.05, 1.30 ± 0.07, 1.20 ± 0.07, and 1.10 ± 0.10) at 50, 100, 150, and 250 ms intervals tested, particularly at the shorter intervals (*p* < 0.01; one-way ANOVA, Figure 2). After CS washout, the ratio value returned to control conditions (1.50 ± 0.06, 1.32 ± 0.07, 1.17 ± 0.08, and 1.12 ± 0.08 at 50, 100, 150, and 250 ms intervals).  Figure 3 shows the time-course of facilitation of fEPSP evoked by paired pulses at 50 ms intervals and applied every 15 s. As indicated above, CS (300 *μ*M) induced a partial reduction of the first fEPSP evoked with no effect on the second fEPSP. The different effects on both signals induced an increase of paired-pulse facilitation of 15.63% at 50 ms intervals. CS-induced depression of fEPSP seems to be caused by decreasing glutamate release probability, as shown by the results of these paired-pulse experiments.

### 3.3. Chondroitin Sulfate Enhances Intrinsic Excitability

**Figure 4:**
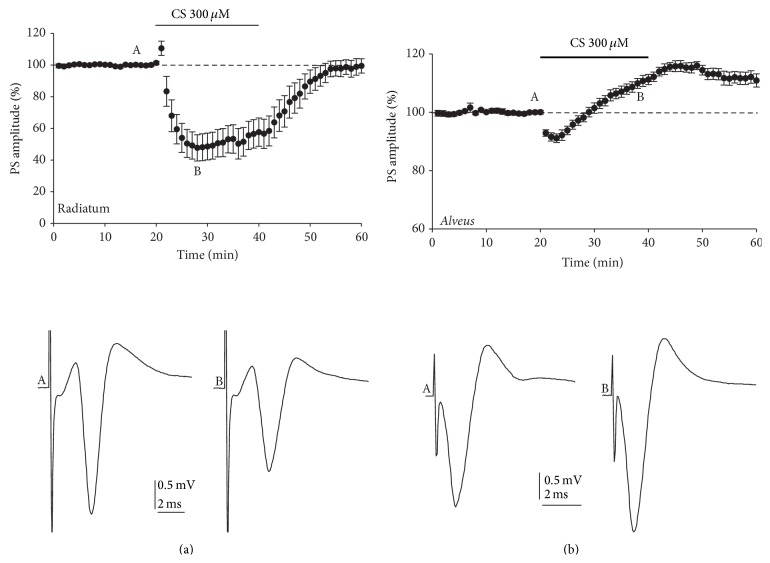
Variation in the amplitude of synaptic evoked population spike responses following CS treatment. (a) The time-courses show average population spike (PS) amplitudes evoked orthodromically by stimulation of* stratum radiatum* that reveal that CS induces a drastic depression of their amplitude in a reversible manner. Bottom panels show representative PS taken at the times indicated by the letters A (control) and B (CS). (b) Time-courses of average PS amplitudes evoked antidromically by stimulation of* stratum alveus* and how CS was able to enhance their amplitude in a reversible manner. As indicated above, the original traces show representative population spike taken at the times indicated by the letters A (control) and B (CS). The graph plots data were normalized to the mean value of the 20 min control period and expressed as mean ± SEM/min (*n* = 6–11 slices).

The results presented above indicate that CS reduces excitatory synaptic input to CA1 pyramidal cells, but they do not tell us how CS affects the pyramidal cell output. To answer this question we explored how CS affected the population spikes (PS) in* stratum pyramidale*, evoked by either the orthodromic stimulation of* stratum radiatum* or the antidromic stimulation of* stratum alveus* (Figure 4). After a period of baseline recording, CS (300 *μ*M) caused a progressive decrement in orthodromically induced PS amplitude reaching a minimum of 36.1 ± 6.7% (2.4 ± 0.3 mV in control and 1.31 ± 0.3 mV after 15 min of CS application, resp.). After drug washout, PS amplitude slowly recovered to baseline values (Figure 4(a)).

Interestingly, when the PS was antidromically evoked by stimulating the* stratum alveus*, which is formed by axons of CA1 pyramidal neurons, after a short period of PS depression (8.21 ± 1.1% at 2–4 min of application), CS caused a progressive increment in antidromically induced PS amplitude that reached a maximum of 110.9 ± 1.7% (2.35 ± 0.83 mV in control and 2.77 ± 0.79 mV after 15 min CS application, resp.). After CS washout, very low recovery of basal PS amplitude was observed (Figure 4(b)).

**Figure 5:**
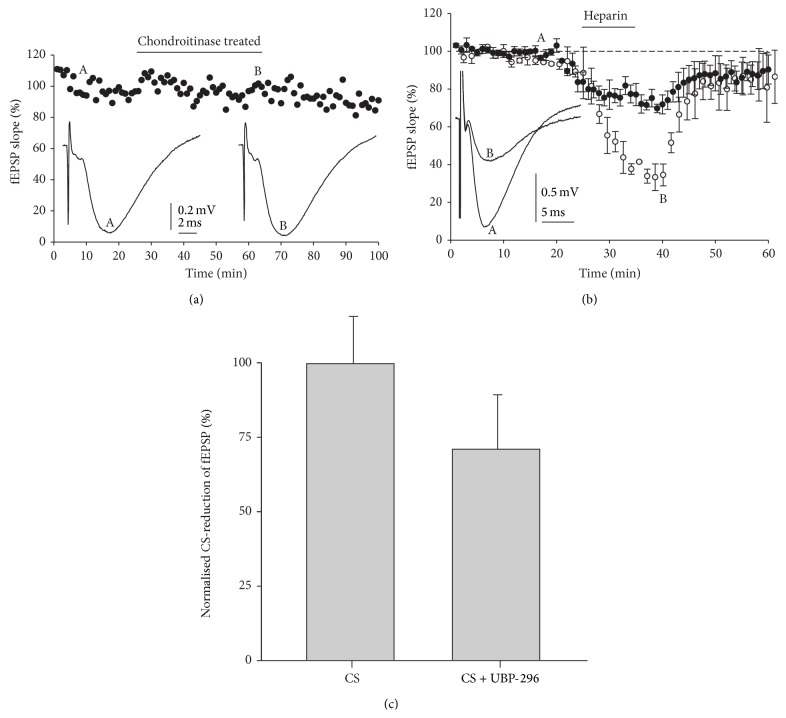
Effects of chondroitinase ABC, heparin, and UBP-296 on synaptic transmission. (a) Time-course of fEPSP slope recorded in the presence of CS (300 *μ*M) treated with chondroitinase during the time indicated by the top horizontal bar. Insets A and B represent the original traces of fEPSPs in the points A and B of the time-course curve. (b) fEPSP recorded in the presence of heparin (100 *μ*M, close circle; 300 *μ*M, open circle). Insets represent the original traces in the points A and B of the time-course curve (heparin 300 *μ*M). (c) Normalised averaged depression of fEPSP observed after application of 300 *μ*M CS in control slices and others pretreated with UBP-293 (30 *μ*M). The bars represent the mean ± SEM of those fEPSPs recorded during the 35 minutes of drug application. Note that no statistical differences were observed. (*n* = 11 slices).

Chondroitinase ABC (50 mU/mL) was used to catalyze the elimination of disaccharides units from polysaccharides containing (1–4)-*β*-D-hexosaminyl and (1–3)-*β*-D-glucuronosyl or (1–3)-*α*-L-iduronosyl linkages [47]; that is, it acts on chondroitin sulfate. In this set of experiments we evaluated whether synaptic depression exerted by CS is due to CS chains or other components of their biologically derived CS. Under treatment with chondroitinase, CS was unable to mimic the effects exerted by CS described above. After a 20 min period of stable fEPSP baseline, chondroitinase treated CS was applied in the perfusion liquid during 50 min, causing no modification of fEPSP (Figure 5(a)).

On the other hand, to study if the effects above reported are specific for CS, we have tested if heparin modified the fEPSP. As mentioned before, only a single concentration of heparin was applied to each individual slice. Once a stable fEPSP baseline was established for 20 min, heparin was applied in the perfusion liquid during the time indicated by the top horizontal bar causing a fast dose-dependent reduction of fEPSP. When heparin was washed out, synaptic responses returned to baseline values in about 5 min (Figure 5(b)). At concentration of heparin (100 and 300 *μ*M), the fEPSP showed a maximal depression of 24.8 ± 3% and 63.7 ± 5%, respectively (*n* = 3-4). So, this effect on synaptic transmission seems to be induced by several types of glycosaminoglycans.

### 3.4. The Blockade of Kainate Receptors Did Not Prevent CS-Induced fEPSP Reduction

In a previous paper we have reported that CS induces Na^+^ inward current and that AMPA/kainate receptor blockers (NBQX and CNQX) fully inhibited the CS effects, while selective blockers of NMDA receptors did not [44]. In this context, we hypothesized that CS induced the inactivation of AMPA receptors; in this manner, the fEPSP, recorded later in the* stratum radiatum* of CA1, evoked by the electrical stimulation of Schaffer collaterals, is lower than the signal recorded before CS application. However, the fEPSPs recorded in our experiments were mainly mediated by the activation of AMPA receptors. Thus, the pharmacological blockade of these receptors to prevent the CS effect described before and so, to demonstrate this hypothesis, is unlikely.

Next, we sought to elucidate whether kainate receptors were involved in the CS-induced reduction of fEPSP. To this end, we bath-applied a selective antagonist of kainate receptors, UBP-296 [48]. As described before, bath application of CS (300 *μ*M, for 20 min) caused a reduction in fEPSP; after 2-3 min of drug washout, the fEPSP recovered to baseline control values. To further evaluate the role of kainate receptors on the CS effect described here, we applied CS in the presence of selective blocker of this receptor type, UBP-296 (30 *μ*M). These antagonists were perfused 20 min before the application of CS (300 *μ*M, for 20 min). UBP-296 shortly reduced fEPSPs by itself (14.9 ± 2.5%) but did not prevent fEPSP reduction caused by CS (Figure 5, *p* > 0.05 when compared with the reduction caused by CS alone).

### 3.5. Chondroitin Sulphate Enhances Induction of LTP

**Figure 6:**
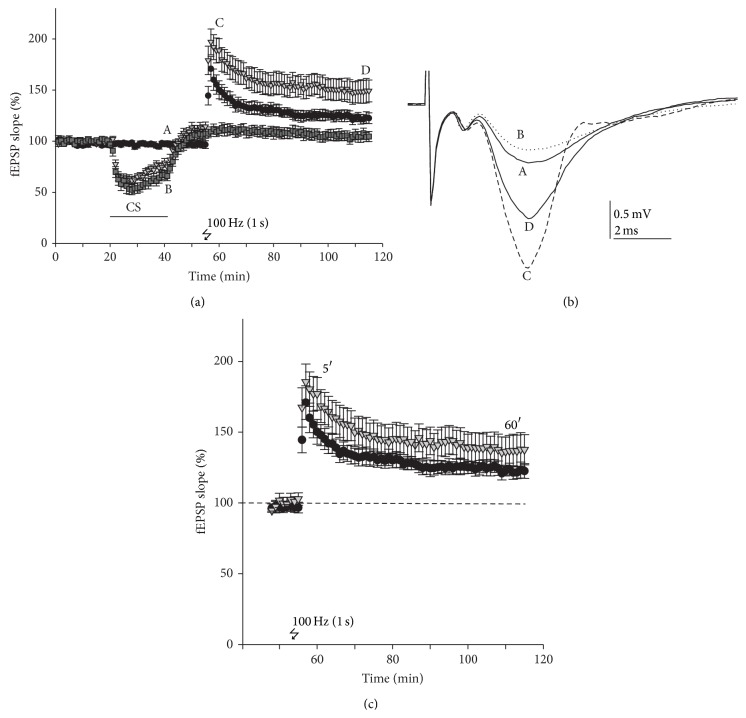
Chondroitin sulfate enhanced short LTP in hippocampal slices. (a) Averaged time-course in control conditions (circle symbol) and CS-treated slices, with (triangle symbol) or without (square symbol) a brief period of tetanization (100 Hz, 1 s). Data were normalized to the mean value of the 20 min control period and expressed as the mean ± SEM/min (*n* = 6–9 slices). (b) Original traces showing representative fEPSP taken at the times indicated by the letters A (control), B (after CS perfusion), C (CS-treated slice after 2 min tetanisation), and D (CS-treated slice after 60 min tetanisation). (c) Time-course of normalised LTP. The baseline recorded before tetanus was considered as 100%.

A brief period of tetanization (100 Hz, 1 s) applied to the Schaffer collateral-commissural fibbers of the CA1 region produced a robust increase in the strength of synaptic transmission (LTP) that persisted for at least 60 min. As previously indicated, CS application (300 *μ*M) produced an average depression of fEPSPs of 23.72 ± 3.8%; this effect was reversible after washout of the drug. To determine if CS could affect the induction of LTP, CS was perfused after a stable baseline and high frequency stimulation (HFS; 100 Hz, 1 s) was applied in the presence of CS. In the slices where LTP was induced in the absence of CS, there was a sharp increase in fEPSP slope (150.2 ± 8.7% of baseline measured immediately after tetanic stimulation) that gradually decreased during the posttetanic potentiation and remained at 122.6 ± 4.3% of baseline after 60 min of HFS. When the tetanic stimulation was applied in slices pretreated with CS, a sharp increase in fEPSP slope was also produced (189.3 ± 11.9% of baseline); this augmented signal remained at 148.8 ± 11.1% of baseline after 60 min of tetanic pulse application (Figure 6(a)). A comparison of the means of the fEPSP slopes recorded 5 min after the tetanic stimulus showed a statistically significant increment of the potentiation showed a statistically significant increment of the potentiation conditions (*p* < 0.05, *n* = 8). However, comparing the fEPSP slopes recorded at the end of the posttetanic period (60 min) showed no statistically increment in the final potentiation (122.6 ± 4.3% and 148.8 ± 11.1% induced in control and CS-pretreated slices). So, we can conclude that CS potentiates the induction of LTP but it does not modify its maintenance. When CS was applied after tetanisation, an enhancement of signal was observed after CS washout during approximately 15–20 min; after this time, as shown above, no statistical differences were observed when compared with control (data not shown). This potentiation after CS application was associated with the nonpermanent rebound observed after CS washout (increase over baseline).  Figure 6(c) shows a time-course of LTP recording normalized to the baseline after CS washout, so this rebound was not considered, and now it is easier to appreciate the fact that CS enhances the induction of LTP but not its maintenance.

## 4. Discussion

Due to the complexity and heterogeneity of CS, the precise structure of CS with biological activity and their underlying molecular mechanisms of action are poorly understood. In the present work we have studied the effects exerted by synthetic CS on evoked synaptic transmission, neuronal excitability, and neuronal plasticity in slices of rat hippocampal neurons. CS caused the following: (i) a reversible depression of fEPSPs in a concentration-dependent manner; (ii) a strong blockade of the population spike amplitude when it was mediated by synaptic transmission but not when it was antidromically evoked; in this last case a potentiation was observed; (iii) an enhancement of paired-pulse facilitation ratio; and (iv) an increase in LTP magnitude. So, our results provide evidence that CS can modulate neuronal communication, synaptic transmission, and neuronal plasticity.

### 4.1. Chondroitin Sulphate Depresses Synaptic Transmission and Alters Neuronal Excitability

In this study we have shown that CS produces a fast, pronounced, and reversible depression of fEPSP in a concentration-dependent manner. The molecular mechanisms involved in the inhibitory effects of CS are not clear. They could be related to a direct interaction with a CS-GAG receptor [25, 26]. This depression may be linked to the capability of CS to modulate the *β*-1 integrin pathway. Recently, some authors have reported that CSPGs restrict spine dynamics and motility through this way [49]. Curiously, this integrin is linked to AMPA receptors and so it is involved in mediating transmission. *β*-1 integrin KO mice showed a drastic reduction in fEPSP at the CA3–CA1 synapse [50]. In addition, in primary culture of hippocampal neurons we have recently reported that CS induces Na^+^-dependent inward whole-cell current and that blockers of AMPA receptors inhibited the CS effects; in contrast, the blockade of NMDA receptors did not modify those effects [44]. With these considerations, we hypothesize that CS induces a partial inactivation of AMPA receptors; in this manner, the latter fEPSP recorded in the* stratum radiatum* of CA1 evoked by the electrical stimulation of Schaffer collateral is lower than the signal recorded before CS application. We noted that CS-induced fEPSP depression was not due to an effect of CS on axon recruitment as inferred from the lack of significant changes observed in the FV amplitude. In spite of these considerations, we cannot discard that the depression exerted by CS may also be due to a direct interaction with the component involved in synaptic transmission or mediated by inducing an increment in the excitability of hippocampal interneurons [25]. Additionally, we would like to point out that some possible discrepancies with other studies may be linked to the specific sulfation sequence chains used, because this feature determines the CSPG functions [29–32, 51].

Here we have found that CS mediated the depression of cellular excitability when population spike was evoked by orthodromic stimulation of* stratum radiatum* fibbers, as expected from the depression of fEPSP elicited by CS. However, our results also evidenced that CS produced a potentiation of neuronal excitability when population spikes were evoked by antidromic stimulation of *alveus* fibbers, in other words, when action potential was not mediated by synaptic transmission. We noticed that this potentiation of population spikes excitability did not develop epileptogenic-like behaviour. The molecular mechanism underlying the CS-modulation of neuronal excitability is also poorly understood. However, we think that the molecular mechanisms underlying these effects may be associated with the control of ions involved in the generation, propagation, and termination of action potential, as reported in neocortex and hippocampal slices were CS-GAGs removal shows an increase in the rate of Ca^2+^ diffusion [52]. Additionally, this idea is compatible with our previous paper where CS produced Na^+^-dependent inward whole-cell current; such current causes cell depolarization and [Ca^2+^]_c_ transient in primary cultures of hippocampal neurons [44].

### 4.2. Effects of Chondroitin Sulfate on Neuronal Plasticity

In spite of CS-induced fEPSP depression, associated with a possible inactivation of AMPA receptors as indicated above, CS also modulates the glutamate release probability as inferred from the significant changes observed with the paired-pulse facilitation paradigm. This enhancement of facilitation may be associated with the property of CS to evoke an elevation of the [Ca^2+^]_c_ [44, 53].

As a result of this modulation of neuronal transmission, we have observed that CS also modifies LTP induction. One of the primary events for induction of LTP is the postsynaptic Ca^2+^ concentration that promotes the incorporation of additional AMPA receptors into the postsynaptic sites [54]. Additionally, components of the extracellular matrix affect ligand binding [37], channel kinetics, and Ca^2+^ permeability of AMPA receptors [54]. Pyramidal cells mostly express Ca^2+^- impermeable AMPA receptors [55, 56]. It has been reported that CS actions are mediated by its interaction with Ca^2+^-impermeable AMPA receptors [44] and that these receptors mediate excitatory synaptic transmission and play a key role in hippocampal LTP.

Thus, the neuronal transmission of the majority of synapses is influenced by extracellular matrix through interactions with their specific receptors. The application of hyaluronidase in hippocampal slices suppresses L-type calcium currents and reduces Ca^2+^ transients in postsynaptic dendrites or spines and abolishes an L-type-mediated component of LTP [38]. After removal of hyaluronic acid, the AMPA receptors diffusion is facilitated and the level of paired-pulse depression under conditions of elevated release probability is reduced [38, 41]. It has been reported that chondroitinase ABC impairs e-LTP [42] and mice deficient in brevican or neurocan show impaired e-LTP and l-LTP, respectively [43].

### 4.3. Potential Physiological Role of Chondroitin Sulphate in the Modulation of Neurotransmission

CS-GAG degradation opens up spaces for axonal growth and the formation of new neuronal contacts [57, 58]. In addition to this structural role, a more pharmacological profile of these degradation products has been suggested [59]. This may be the case for CS that modulates synaptic transmission and processes such as LTP.

The biological activity of CS is defined by the sulfation pattern of repeating disaccharide units [60]. Our CS from Bioibérica consists of highly purified chondroitin sulfate of bovine origin in a concentration not less than 98%; however, we cannot discard that the effects here reported may be, at least in part, due to compounds included in the 2% remaining. Our product consists of a mixture of CS sulfated in positions 4 (62%), 6 (32%) or unsulfated (6%) on the N-acetyl-D galactosamine group. Other authors have reported that only CS with desulfation in the 4 and 6 position of N-acetyl-D galactosamine group is critical for its biological activity [61]. However, other studies also point out that in spite of the sulfation in the 6 position of N-acetyl-D galactosamine group a 2 position sulfation of glucuronic acid for biological activity is also necessary [34, 62]. We have also tested a commercial form of chondroitin with sulfation in the 4 position that did not mimic the effect obtained by CS from Bioibérica.

In conclusion, we have found that in rat hippocampal slices CS depressed fEPSPs reduced the population spike amplitude evoked by orthodromic stimulation and augmented paired-pulse facilitation as well as LTP. These data support the hypothesis that degradation of extracellular matrix could release free CS that will then exhibit its effects on enhanced Ca^2+^ signaling and Ca^2+^-dependent neuronal plasticity. This remodeling of perineuronal nets contributes to brain plasticity and hence to regulating cognition, tissue repair, cell migration, or axon regrowth [18].
